# Malnutrition prevalence and precision in nutritional care differed in relation to hospital volume – a cross-sectional survey

**DOI:** 10.1186/1475-2891-8-20

**Published:** 2009-05-08

**Authors:** Albert Westergren, Christine Wann-Hansson, Elisabet Bergh Börgdal, Jeanette Sjölander, Rosmarie Strömblad, Rosemarie Klevsgård, Carolina Axelsson, Christina Lindholm, Kerstin Ulander

**Affiliations:** 1 Research and Development Unit, Central Hospital Kristianstad, Kristianstad, Sweden; 2 School of Health and Society, Kristianstad University College, Kristianstad, Sweden; 3 Faculty of Health and Society, Malmö University and Malmö University Hospital, Malmö, Sweden; 4 Department of Emergency Medicine, Malmö University Hospital, Malmö, Sweden; 5 Department of Clinical Nutrition, Lund University Hospital, Lund, Sweden; 6 Hospital Management, Blekinge Hospital, Karlskrona, Sweden; 7 Hospital Management, Lund University Hospital, Lund, Sweden

## Abstract

**Background:**

To explore the point prevalence of the risk of malnutrition and the targeting of nutritional interventions in relation to undernutrition risk and hospital volume.

**Methods:**

A cross-sectional survey performed in nine hospitals including 2 170 (82.8%) patients that agreed to participate. The hospitals were divided into large, middle, and small sized hospitals. Undernutrition risk and overweight (including obesity) were assessed.

**Results:**

The point prevalence of moderate/high undernutrition risk was 34%, 26% and 22% in large, middle and small sized hospitals respectively. The corresponding figures for overweight were 38%, 43% and 42%. The targeting of nutritional interventions in relation to moderate/high undernutrition risk was, depending on hospital size, that 7–17% got Protein- and Energy Enriched food (PE-food), 43–54% got oral supplements, 8–22% got artificial nutrition, and 14–20% received eating assistance. Eating assistance was provided to a greater extent and artificial feeding to a lesser extent in small compared to in middle and large sized hospitals.

**Conclusion:**

The prevalence of malnutrition risk and the precision in provision of nutritional care differed significantly depending on hospital volume, i.e. case mix. It can be recommended that greater efforts should be taken to increase the use of PE-food and oral supplements for patients with eating problems in order to prevent or treat undernutrition. A great effort needs to be taken in order to also decrease the occurrence of overweight.

## Background

Nutritional screening is important in identifying persons who require treatment, as malnutrition is an under-recognised and under-treated condition [1-3]. Nutritional screening, assessment and treatment is emphasised in a resolution from the Council of Europe [4] and Swedish hospitals have been found to have difficulties in living up to the recommendations [5]. However, knowledge about the correspondence between the findings from nutritional screening and the actual provision of treatment is sparse. From a general perspective, knowledge about the prevalence of risk of malnutrition, i.e. undernutrition risk and overweight (including obesity), is important as it describes the magnitude of these problems and has implications for allocating health care resources for helping patients to remain or become well nourished.

Several factors influence the prevalence of risk of malnutrition. Among these are type of patients, i.e. case mix, within the different hospital settings and also the methods used to measure undernutrition or overweight. This can make it difficult to compare between different hospitals. To give some examples, it was found that 27% of the patients in middle and small sized hospitals (< 500 beds) were at risk of undernutrition (which means at least two of: unintentional weight loss, low Body Mass Index (BMI) and eating difficulties) [6], while in a teaching hospital it was 27% (based on anthropometry) or 46% (based on Subjective Global Assessment (SGA)) that were at undernutrition risk [7]. In a national screening in the Netherlands, 26% of the patients were found to be undernourished (weight loss) including all types of hospitals [8]. In a British study, 26% were at medium or high risk of undernutrition in smaller hospitals (< 1000 beds) and 38% in larger hospitals (> 1000 beds, Malnutrition Universal Screening Tool) [9]. The opposite was found in a nationwide German hospital study were 27% were malnourished according to SGA, and more were undernourished in smaller (37%) than in larger hospitals (20%) [10]. Oncological, gastrointestinal and lung diseases are associated with the highest prevalence of undernutrition [8].

It is not only undernutrition risk that is a problem in hospitals, but also overweight. The prevalence of overweight has also been presented previously, and in the British survey 52% were found to be overweight (BMI > 25 kg/m^2^) [9]. In another study [6], the prevalence of overweight in middle and small sized hospitals was found to be 39% (BMI =/> 25 if </= 69 yrs, BMI =/> 27 if =/> 70 yrs). The studies referred to indicate that there are differences in the prevalence of risk of malnutrition in relation to type of hospital and the case mix therein. In addition, the prevalence found in different studies varies due to differences in definitions and the use of different screening tools [11]. Thus, it seems worthwhile to explore the prevalence of undernutrition risk as well as overweight using the same criteria (one screening tool), in relation to hospital volume.

Besides exploring the prevalence of undernutrition risk and overweight, it is important to gain knowledge about how well nutritional interventions are targeted to those at undernutrition risk. This is especially interesting with regard to patients with energy problems (who eat little, stop eating due to tiredness rather than to having satisfied their hunger, eat slowly and/or have poor appetite), especially as these types of problems have been found to have a negative impact on nutritional status and are related to the provision of protein- and energy enriched food and oral supplements [12,13].

The aim of this study was to explore the prevalence of risk of malnutrition among persons in Swedish large, middle and small sized hospitals. In addition, the aim was to explore how well nutritional interventions are targeted towards patients at undernutrition risk.

## Methods

### Study Context

Swedish hospitals can be divided into regional hospitals, central general hospitals and general hospitals. Regional hospitals (or teaching hospitals, in this study labelled large sized hospitals with > 500 beds) have resources for the county, region, and in most cases also for high speciality national health care. Central general hospitals (in this study middle sized hospitals with 200–500 beds) are responsible for covering the whole county population's need for health care. The general hospitals (in this study small sized hospitals with < 200 beds) have the responsibility for a limited part of the county population's need for health care. The regional hospitals and the central general hospitals can provide 24-hour emergency admittance and have surgery departments, intensive care and radiology departments available on a 24-hour basis. These hospitals also have physicians within certain specialities (surgery, orthopaedics, medicine, gynaecology, radiology and anaesthesiology) on duty on a 24-hour basis.

### Sample

The study was performed in 2007 during one single day in nine hospitals with a total coverage area of 1 197 500 inhabitants (Table 1).

**Table 1:** Descriptions of included hospitals and the total coverage area of habitants.

	Hospital number	Coverage population
**Large Sized Hospitals (n = 2)**	1	274 000
Regional hospitals with > 500 beds	2	250 500
		
**Middle Sized Hospitals (n = 3)**	3	103 500
Central General Hospitals with 200–500 beds	4	163 500
		
	5	150 000
		
**Small Sized Hospitals (n = 4)**	6	93 000
General Hospitals < 200 beds	7	70 000
	8 and 9	93 000
**Total Coverage Population**		1 197 500

The inclusion criteria were that all adult in-hospital patients (18 years or over) registered at the ward between 7 a.m. and 9 p.m. should be asked for participation. No intensive, out-patient or delivery units participated. Out of 2 620 patients, 2 170 (82.8%) agreed to participate. In large sized hospitals (n = 2) 1 197 (84.0%) out of 1 426 patients participated. In middle sized hospitals (n = 3) 669 (81.2%) out of 824 patients participated. In small sized hospitals (n = 4) 304 (82.2%) out of 370 patients participated. In the total sample, there were no significant differences regarding age (p-value = 0.086) and gender (p-value = 0.331) between those included (n = 2 167) and drop-outs (n = 445).

### Procedure

Nursing students, clinical tutors and registered nurses and dieticians got training and education in how to collect the data and were then responsible for the data collection. Data was collected through measures of height and weight, interviews and observations of patients during mealtimes. The data collection was preceded by gaining informed consent.

### Protocol and Definitions

The protocol contained three parts. The first part included background data about the patients. The second part included data about nutrition and eating difficulties. The third part contained information about nutritional support.

Risk of malnutrition in this study includes both undernutrition risk and overweight. Height and weight were measured using the standard equipment available at the particular units. Moderate/high undernutrition risk was defined as the occurrence of at least two of: involuntary weight loss, BMI below limit (BMI < 20 if </= 69 yrs, BMI < 22 if >/= 70 yrs), eating difficulties according to Minimal Eating Observation Form – Version II (MEOF-II) [13] based on Swedish recommendations for detecting undernutrition risk [6,14]. Information about unintentional weight loss was gained from the patient or estimated from previous weight.

Minimal Eating Observation Form – Version II (MEOF-II) includes three components of eating. **Ingestion **includes "manipulation of food on the plate", "transport of food to the mouth" and "sitting position". **Deglutition **includes "ability to chew", "manipulation of food in the mouth" and "swallowing". **Energy **includes "alertness", "appetite" and "eating < 3/4 of served food" [13].

Overweight was graded based on BMI (if </= 69 yrs: BMI =/> 25: if >/= 70 yrs: BMI =/> 27) and so was obesity (if </= 69 yrs: BMI 30–39: if >/= 70 yrs: BMI 32–41) and severe obesity (if </= 69 yrs: BMI =/> 40: if >/= 70 yrs: BMI =/> 42) [6].

Protein- and Energy Enriched food (PE-food) is food that is smaller in volume than the regularly served meals but has the same or higher content of protein and energy compared to the ordinary hospital food on the menu. "Supplements" include oral nutritional supplements such as protein and energy drinks given in addition to and chiefly between the main meals. Supplements do not include pharmacological therapy or drug supplement with multivitamin and mineral pills. Artificial nutrition includes enteral feeding (nasogastric tube, gastrostomy or jejunostomy) and parenteral feeding (via a peripheral or central vein) given alone or as a supplement to oral intake, and pre- and/or postoperative artificial nutrition with glucose or sodium chloride solutions. Eating assistance includes both partial (buttering bread, cutting food, only helping with beverage) and total assistance.

### Analysis

Parametric and non-parametric statistics were used depending on the level of data and based on unpaired comparisons between two or three groups. The following tests were applied: Chi-square test, Kruskal Wallis test, Mann Whitney *U*-test, and one way ANOVA (with post-hoc analysis by Bonferroni correction). The level of statistical significance was set at P-value < 0.05. When multiple comparisons were made (going from three to two group comparisons) a reduced p-value of < 0.017 was used to avoid mass significance (type I or alpha error) [15]. Analyses were performed using SPSS version 16.0 (SPSS Inc., Chicago, IL, USA).

### Ethics

The ethics for conducting scientific work was followed. This study was approved at each hospital. The patients were asked for informed consent. Both verbal and written information was given and patients were guaranteed anonymity, i.e. no personal identification number or names were collected. As the study was a part of an overall quality development project, no formal approval by an ethical committee was required, according to the Swedish Act concerning the Ethical Review of Research Involving Humans [16].

## Results

There were significant differences between the three hospital samples regarding age. Patients in large sized hospitals were younger than those in middle and small sized hospitals, and those in middle sized hospitals were younger than those in small sized hospitals. Large and middle sized hospitals also had significantly more patients under surgical treatment compared to small sized hospitals. In addition there were significantly more psychiatric patients in small compared to in middle and large sized hospitals, and in middle compared to large sized hospitals. Correspondingly there were significant differences in diagnosis between the three samples. For instance, middle sized hospitals had more patients with gastrointestinal diseases, while the small sized had more patients with cardiovascular, psychiatric and orthopaedic diseases. There were significantly more patients with oncological diseases in large and middle sized than in small sized hospitals (Table 2).

**Table 2:** Characteristics of patients and divided according to the size of hospital.

	Hospitals			
	Large sized	Middle sized	Small sized	
	n = 1197	n = 824	n = 370	P-value
Age, mean (SD)	66 (18)	69 (16)	70 (16)	0.001 ^a, b, c^
Age group				< 0.005 ^a, b^
< 70 years, %	51.7	45.0	39.9	
≥ 70 years, %	48.3	55.0	60.1	
Gender, %				0.580
Men	49.5	48.0	46.5	
Women	50.5	52.0	53.5	
Distribution of patients within some specialities, %				
Medicine	36.9	41.0	33.2	0.092
Surgery	16.8	17.0	10.5	0.022 ^b, c^
Orthopaedics	8.6	11.1	13.8	0.018 ^d^
Psychiatry	5.3	2.6	8.9	0.001 ^a, b, c^
Distribution of patients according to some diagnosis categories, %				
Pulmonary	6.1	11.6	9.8	< 0.0005 ^a, b^
Cardiovascular	19.0	22.4	30.6	< 0.0005 ^b, c^
Infectious	9.9	7.3	4.0	0.003 ^b, c^
Gastrointestinal	13.4	20.3	3.4	< 0.0005 ^a, b, c^
Neurological	4.3	5.2	2.4	0.139
Orthopaedic	10.8	7.8	29.0	< 0.0005 ^a, b, c^
Psychiatric	6.1	3.1	11.1	< 0.0005 ^a, b, c^
Oncological	28.5	24.2	7.3	< 0.0005 ^b, c^

Missing values in < 5%. ANOVA, Chi-square test

^a ^first group differs compared to second

^b ^first group differs compared to third

^c ^second group differs compared to third group

^d ^not significant in post hoc analysis

Eating difficulties were significantly more common among patients in large compared to middle and small sized hospitals, and in middle compared to small sized hospitals. Unintentional weight loss and undernutrition risk were significantly more common in large compared to middle and small sized hospitals. In large sized hospitals 38.2% of patients were overweight (including obesity) and in middle and small sized hospitals it was 42.6% and 42.2% respectively (no significant difference) (Table 3).

**Table 3:** Point prevalence of risk of undernutrition (UN) and overweight among the studied patients.

	Hospitals			
	Large sized	Middle sized	Small sized	P-value
	n = 1197	n = 824	n = 370	
Criteria for UN-risk, %				
Eating difficulties according To MEOF-II	58.2	50.4	42.5	< 0.0005 ^a, b, c^
Low BMI	22.3	19.9	17.2	0.145
Unintentional weight loss	40.2	34.1	27.2	0.001 ^a, b^
Fulfilling UN risk criteria, %				< 0.0005 ^a, b^
No criteria – no UN risk	31.0	35.2	47.3	
One criteria – low UN risk	35.0	38.6	31.1	
Two criteria – moderate UN risk	26.4	21.1	17.7	
Three criteria – high UN risk	7.6	5.1	3.9	
Overweight, %				0.102
No overweight	61.8	57.4	57.8	
Grade 1, overweight	26.0	27.0	31.0	
Grade 2, obesity	11.2	14.8	11.2	
Grade 3, severe obesity	1.0	0.8	0.0	

MEOF-II = Minimal Eating Observation Form – Version II. Missing values in < 10%. Chi-square test, Kruskal Wallis test, Mann Whitney *U *test

^a ^first group differs compared to second

^b ^first group differs compared to third

^c ^second group differs compared to third group

Having ingestion and deglutition difficulties was significantly more common among patients in large compared to small sized hospitals. Energy problems were more common among patients in large sized compared to in middle and small sized hospitals, and in middle compared to small sized hospitals (Table 4).

**Table 4:** Distribution (%) of eating difficulties divided according to the size of each hospital.

	Hospitals			
	Large sized	Middle sized	Small sized	P-value
	n = 1197	n = 824	n = 370	
Eating difficulties				
Ingestion	12.2	9.0	8.0	< 0.029 ^b^
Deglutition	20.5	14.0	15.3	0.001^b^
Energy	50.3	43.5	34.2	< 0.0005 ^a, b, c^

Missing values in < 5%. Chi-square test

^a ^first group differs compared to second

^b ^first group differs compared to third

^c ^second group differs compared to third group

There were no significant differences between hospitals in the precision of nutritional interventions in relation to undernutrition risk despite for artificial feeding and in the provision of eating assistance. More patients at no/low risk of undernutrition got artificial nutrition in large compared to small sized hospitals and in middle compared to small sized hospitals. More patients in small sized hospitals were provided eating assistance compared to in middle and large sized hospitals. Among those with moderate/high risk of undernutrition, significantly more patients got artificial feeding in large sized and middle sized hospitals than in small sized hospitals (Table 5).

**Table 5:** The precision (%) in the nutritional care for patients at no/low risk and for patients at moderate/high risk of undernutrition.

	Hospitals			
	Large sized	Middle sized	Small sized	P-value
**At no/low risk of undernutrition**	n = 760	n = 465	n = 222	
PE-food	5.3	4.0	1.9	0.122
Oral Supplement	16.3	19.0	13.5	0.182
Artificial nutritional (AN) support	15.0	11.3	3.3	< 0.0005 ^b, c^
Pre- and/or postoperative AN	5.4	5.2	1.8	0.077
Eating Assistance	5.3	6.4	11.7	0.011 ^b, c^
**At moderate/high risk of undernutrition**	n = 392	n = 165	n = 61	
PE-food	14.4	16.6	6.8	0.181
Oral Supplement	44.8	54.1	43.3	0.115
Artificial nutritional support	22.5	22.1	8.3	0.040 ^b, c^
Pre- and/or postoperative AN	6.1	9.7	3.3	0.160
Eating Assistance	18.3	14.4	20.3	0.593

Missing values in < 15%. Chi-square test

^a ^first group differs compared to second

^b ^first group differs compared to third

^c ^second group differs compared to third group

Patients with energy problems in small sized hospitals got less artificial feeding, less pre- and/or postoperative artificial nutrition and more eating assistance than patients in large and middle sized hospitals. Other than that, there were no significant differences between hospitals in the targeting of nutritional care in relation to energy problems (Table 6).

**Table 6:** The precision (%) in the nutritional care in relation to patients with energy problems (eat little, stop eating due to tiredness rather than to having satisfied their hunger, and eat slowly)

	Hospitals			
	Large sized	Middle sized	Small sized	P-value
**Energy problems**	n = 599	n = 276	n = 103	
PE-food	12.6	12.1	5.9	0.159
Oral Supplement	38.9	43.4	37.6	0.407
Artificial nutritional (AN) support	21.6	23.2	11.0	0.031 ^b, c^
Pre- and/or postoperative AN	6.2	10.1	1.9	0.011 ^b, c^
Eating Assistance	15.7	16.5	28.0	0.019 ^b, c^

Missing values in < 15%. Chi-square test

^a ^first group differs compared to second

^b ^first group differs compared to third

^c ^second group differs compared to third group

## Discussion

The results of this study clearly demonstrate that there are significant differences in the prevalence of undernutrition risk in relation to hospital volume. Eating assistance is provided to a greater extent and artificial feeding to a lesser extent in small compared to in middle and large sized hospitals. Other than that, there is no difference in the precision of provision of nutritional care.

Prevalence studies always reflect a snapshot of reality and must therefore be interpreted with care. In this large survey many persons were involved in the data collection, which can be seen as a shortcoming of the study. However, all the staff responsible for data collection had got the same education about procedure, screening and how to fill in the study protocol. This method of data collection is very useful when the goal is to reach a large sample using limited resources. In addition, there are gains made for the students and clinical practitioners, such as awareness of research methodology and nutrition and eating difficulties, by involving staff and students in the data collection [17].

The same methodology used in the present study was used in an earlier study in 2005 [6] and the instrument MEOF II for detecting eating difficulties was then slightly modified based on psychometric criteria [13]. However, the combination of unintentional weight loss, low BMI and MEOF II for defining undernutrition risk need to be compared to other validated instruments in future studies.

The prevalence of undernutrition risk found in large sized hospitals cannot automatically be generalised to middle or small sized hospitals due to differences in patient populations. A stepwise decrease, from large sized to small sized hospitals, was found in the number of patients with moderate or high undernutrition risk. The same pattern was found in the British survey, with a higher prevalence of undernutrition risk in large sized hospitals than in smaller hospitals [9]. Such a pattern was expected (but not confirmed) in the German nationwide survey [10]. The researchers stated in the discussion that the prevalence of undernutrition risk was expected to be higher in the larger hospitals, as patients admitted to university hospitals might be more severely sick and thus more prone to malnutrition [10]. However, it has not been demonstrated that the patients (in general) in university hospitals are more sick than those in general hospitals. Instead, it can even be that teaching/university hospitals admit healthier patients than general hospitals and also perform higher volumes of procedures than general hospitals. At least, this seems to be the case in cardiology [18]. However, hypothetically it can be that the complexity of diseases rather than "severity of illness" [19] cause the higher prevalence of undernutrition risk. Also differences in comorbidity may explain the association between hospital volume and outcome, i.e. undernutrition [20]. The hypothesis about comorbidity/complexity/rarity is supported by the fact that more patients in large sized hospitals in the present study had different types of eating difficulties, and at the same time, they were younger than the patients in middle and large sized hospitals. In addition, it is known that advanced age predisposes to nutritional deficits [10]. If only age and not the characteristics of the case mix were considered as an explanation, one would expect a higher prevalence of undernutrition risk in smaller hospitals. However, in the present study there were many patients with oncological, gastrointestinal and cardiovascular diseases in the large sized hospitals, diagnoses known to involve a high prevalence of undernutrition risk [8,10]. It is difficult to draw any firm conclusions about the reasons for the higher prevalence of undernutrition risk in larger hospitals, but it is likely that the characteristics of the case mix in different hospitals is the cause of this phenomenon. There is a need to further explore the reasons behind the differences in prevalence of undernutrition risk in relation to hospital volume.

Significantly more patients in large and middle sized hospitals got artificial nutrition compared to in small hospitals, while patients in small sized hospitals got more assisted feeding. One explanation could again be that this difference reflects characteristics of the case mix in the different hospitals, or rather the adaptation of treatment due to specific disease characteristics only superficially controlled for in this study. A second and more controversial explanation could be that one is more prone to give artificial feeding to younger patients and feeding assistance to older patients. A third explanation could be that there is a culture in large sized hospitals to use technical solutions (artificial feeding) to a greater extent than in small sized hospitals. This last explanation is supported by the fact that university hospitals use "more procedures" (invasive investigations, treatments) than general hospitals [18,20]. However, in this study one should be careful in interpreting artificial feeding as an intervention decided on due to only undernutrition risk, as there could have been other reasons for this action. More studies are needed that explore the targeting of nutritional interventions towards those needing them most.

Larger hospitals have more patients with eating difficulties than smaller hospitals. Especially energy problems differed in relation to the size of hospital, with more patients having energy problems in large sized hospitals. A difference in the precision of common nutritional interventions (i.e. PE-food, oral supplements) could perhaps have been expected, by means of higher precision in the large sized and more specialised teaching hospitals. No other study has been found (PubMed search, January 2009) that looks specifically at the targeting of PE-food and oral supplements in relation to undernutrition risk. But a previous study [13] found that staffs are good at providing eating assistance for patients with ingestion difficulties, and that these problems do not strongly contribute to undernutrition risk. It has also been found that energy problems are the single most important factor among the eating difficulties that contribute to undernutrition risk [12,13]. A better targeting of PE-food and oral supplements is perhaps the most important step to take, as it is well known that dietary supplementation is beneficial by means of for instance better values in anthropometric measures, decreased hospital stay and mortality [21-26]. In this study, only 6–13% of patients with energy problems (eat little, stop eating due to tiredness, eat slowly) got PE-food and 39–43% of patients got oral supplements. Thus, it can be concluded that staff are less good at targeting these interventions (PE-food, supplements) towards patients with energy problems and that these problems therefore are likely to be among the strongest contributing factors to the development or maintenance of undernutrition risk [13].

Many patients admitted to hospitals are overweight. Between 38% and 43% were found to be overweight. Preventive actions such as information about the risks connected to overweight and the importance of exercise and eating healthy food, and help to overweight persons with losing weight, need to be taken, especially if there are weight-related health problems [27]. Studies have shown that weight-loss therapy improves physical functioning and quality of life, and decreases the medical complications associated with obesity in older persons [27]. However, voluntary weight loss needs to take place under controlled forms in order to not cause loss of bone mass or muscle mass, and not during the acute phase of disease. Thus, information should be given in hospitals and the weight-loss therapy can start after hospital discharge when the health status has stabilised. It is also important to combine weight-loss therapy with physical activities. To sum up, clinical practice needs to focus on both undernutrition and overweight.

## Conclusion

The prevalence of undernutrition risk differs depending on the case mix that in turn is related to the hospital volume. There are no differences in the precision in providing PE-food and oral supplements that are due to the hospital volume, while there are differences in the type of eating difficulties that the patients have. It can be recommended that greater efforts should be taken to increase the use of PE-food and oral supplements, especially for patients suffering from a lack of energy (eat little, stop eating due to tiredness, poor appetite). Also great efforts need to be taken to decrease the occurrence of overweight. Thus the awareness among physicians, nurses and other professionals must be improved about how to increase the precision in provision of nutritional care. This can be done through education in nutritional screening, assessment and treatment and also by implementation of national recommendations in food and nutritional care in hospitals.

## Competing interests

The authors declare that they have no competing interests.

## Authors' contributions

AW was the lead investigator of the study (together with KU). He was involved in the design of the study, providing information to data collectors, analysing data and writing the manuscript. KU, CA, EBB, CWH, and JS were involved in the design of the study, providing information to data collectors and critically revising the manuscript. CL, RK and RS were involved in the design of the study and critically revising the manuscript. All authors besides KU (deceased) approved the final manuscript.
